# Essentials of Materia Medica

**Published:** 1890-01

**Authors:** 


**Essentials of Materia Medica,** Therapeutics and Prescription Writing, arranged in the form of Questions and Answers, prepared especially for Students of Medicine. By HENRY MORRIS, M. D., late Demonstrator, Jefferson Medical College, Philadelphia; Co-Editor Biddle's Materia Medica; Visiting Physician to St. Joseph's Hospital; Fellow College of Physicians, Philadelphia, etc., etc. 12mo, pp. xvi., 250. Philadelphia: W. B. Saunders, 913 Walnut street. 1889.

This book is No. 7 in Saunders's question-compends, and is uniform in style and general appearance with the others of the series. The subjects of which this author treats are presented in a unique and attractive manner, such as cannot fail to impress the young mind and instruct it in a lasting way. Moreover, the author is experienced in teaching, and comprehends the avenues through which instruction must be imparted in order to make it valuable. Three conditions are required to make learning valuable; first, it must be comprehended; second, it must be remembered; and third, it must be applied. This book, in the hands of a well-trained mind, will be found useful in the directions named. Its beautiful make-up, which is a fine specimen of book art, will contribute not a little to make it serve its purpose.

